# Predictive Factors of Recovery after an Acute Lateral Ankle Sprain: A Longitudinal Study

**DOI:** 10.3390/sports9030041

**Published:** 2021-03-18

**Authors:** Philippe Terrier, Sébastien Piotton, Ilona M. Punt, Jean-Luc Ziltener, Lara Allet

**Affiliations:** 1 Haute-Ecole Arc Santé, HES-SO University of Applied Sciences and Arts Western Switzerland, 2000 Neuchâtel, Switzerland; 2 Division of Thoracic Surgery, University Hospitals of Geneva, 1205 Geneva, Switzerland; 3 Division of Orthopaedics and Trauma Service, University Hospitals of Geneva, 1205 Geneva, Switzerland; sebastien.piotton@hirslanden.ch; 4 Department of Surgery and Trauma Surgery, Maastricht University Medical Center and NUTRIM School for Nutrition and Translational Research in Metabolism, Maastricht University, 6229 HX Maastricht, The Netherlands; Ilona.Punt@maastrichtuniversity.nl; 5 Department of Orthopaedics and Research School Caphri, Maastricht University Medical Center, 6229 HX Maastricht, The Netherlands; 6 Hirslanden Clinique La Colline, 1206 Geneva, Switzerland; Jean-Luc.Ziltener@hirslanden.ch; 7 School of Health Sciences, HES-SO Valais Wallis University of Applied Sciences and Arts Western Switzerland, 1950 Sion, Switzerland; Lara.Allet@hevs.ch; 8 Department of Community Medicine, University Hospitals and University of Geneva, 1205 Geneva, Switzerland

**Keywords:** sport injury, ankle injury, recovery prediction, early prognosis, functional score, walking pain

## Abstract

A prominent feature of ankle sprains is their variable clinical course. The difficulty of providing a reliable early prognosis may be responsible for the substantial rate of poor outcomes after an ankle sprain. The aim of the present study was to evaluate the prognostic value of objective clinical measures, pain, and functional scores for ankle sprain recovery. Fifty-two participants suffering from lateral ankle sprain were included. Sprain status was assessed four weeks following injury and included evaluations of ankle range of motion, strength, function, and pain. Seven months following injury, a second assessment classified the patients into recovered and non-recovered groups using ankle ability measures. Following a predictor pre-selection procedure, logistic regressions evaluated the association between the four-week predictors and the seven-month recovery status. Twenty-seven participants (52%) fully recovered and 25 did not (48%). The results of the logistic regressions showed that walking pain was negatively associated with the probability of recovering at seven months (odds ratio: 0.71, 95% CI: 0.53–0.95). Pain four weeks after ankle sprain had relevant predictive value for long-term recovery. Special attention should be paid to patients reporting persistent pain while walking four weeks following sprain to reduce the risk of chronicity.

## 1. Introduction

Lower-extremity joint injury is among the most common consequences of accidents occurring in recreational and athletic activities [1]. A large percentage of such injuries are ankle sprains—that is the stretching, partial rupture, or complete rupture of at least one ligament about the talocrural joint [2]. In the United States (US), ankle sprains occur with an incidence rate of 2.15 to 3.29 per 1000 person each year among the general population [3,4]. Among exposed individuals, such as US military cadets, the incidence reaches as high as 58.4 per 1000 person each year [3]. Likewise, ankle sprains represent 15% of all sport-related injuries [5]. A meta-analysis revealed that court and indoor sports (e.g., basketball, gymnastics, and tennis) pose the highest risk of ankle sprain [6]. Dance is also a problematic activity, with 42% to 97% of dancers injured yearly and ankle sprain prevalence ranging from 14% to 54% [7]. In 2010, one million individuals were treated in US emergency departments for ankle sprain, at a median cost of 1029 USD per case, accounting for significant health care charges [4].

Following an initial assessment (including history, physical and radiographic evaluations), acute treatment for ankle sprains entails anti-edema measures (protection, rest, ice, compression, elevation, (PRICE)), anti-inflammatory medication, and weight-bearing support by means of a brace. Severe sprains may require rigid immobilization (ten days), followed by a transition to semi-rigid external constraints [8,9]. Surgical intervention should be restricted to high-demand patients, such as professional athletes [10] and patients with severe sprains who failed to respond to conservative treatment [11]. Physical therapy usually follows acute-phase treatments [9] and focuses on muscle strengthening, joint mobilization, and neuromuscular training. The aim of rehabilitation is to restore the range of motion (ROM) and proprioception of the injured ankle and to reinstate lower limb strength [12]. A recent meta-analysis concluded that exercise therapy—including coordination, balance, and neuromuscular training—is useful to prevent recurrent ankle sprains [9]. 

Healing times and capacities to achieve full recovery vary among patients recovering from ankle sprain. Some of these patients will return to their daily living activities without limitations within a few days, without having to take part in physical therapy. Others will develop chronic ankle instability (CAI) and chronic pain and will endure sprain sequelae over a period of years after the injury despite intensive rehabilitation. Roughly, one out of every five members of the public who incur an ankle sprain will report chronic problems [13,14]. A systematic review of 31 follow-up studies showed that only 35% to 85% of patients exhibited full recovery within a period of three years following ankle injury [15]. This review also reported that 5% to 33% of ankle injury patients still experienced pain after one year; in addition, as many as 33% of the patients reported at least one re-sprain within a three-year period, relating to the development of CAI [14]. These mixed outcomes cast doubt on the efficacy of the early detection of patients most at risk for long-term complications. These findings also cast doubt on practitioners’ ability to select the appropriate therapeutic approach for a given individual. 

One reason for the non-negligible rate of poor outcomes following ankle sprain is the difficulty inherent in providing a reliable prognosis. For example, structural lesions found by radiological investigation (e.g., MRI) are not related to the persistent complaints of patients [16]. A variable clinical course is a prominent feature of ankle sprains. Therefore, tools that can correctly classify patients according to their risk of developing chronic symptoms are most desired. Indeed, patients most at risk for developing CAI and chronic pain should be treated with intensive exercise therapy [9]. However, a recent evidence-based clinical guideline conceded that “not all factors contributing to the success or failure of rehabilitation are known” and concluded that “further research on prognostic factors is required and may provide additional and more uniform insights” [9].

What kinds of clinical observation may be helpful in characterizing patients at risk of developing complications after ankle sprain? A recent systematic review of nine articles summarized the prognostic factors of recovery following acute lateral ankle sprain [17]. With regard to mid-term recovery (more than 4 months), significant prognostic factors included age, female gender, MRI-visible lesions, resting pain (at three months), and re-sprains (within three months). However, due to the poor quality and methodological heterogeneity of the individual studies, the authors concluded that the associations of these prognostic factors with poor recovery status were largely inconclusive. Nevertheless, this review highlighted the importance of self-reported functional ability and the role of biomechanical factors in recovery. 

The present study is part of an effort to improve the management of patients suffering from lateral ankle sprain. In this context, it is essential to characterize factors that may be associated with an unfavorable sprain evolution to detect patients most at risk of complications. Therefore, the objective of this study was to evaluate the prognostic value of objective clinical measures, self-reported pain, and functional scores acquired four weeks following injury to predict long-term (seven-month) recovery.

## 2. Materials and Methods

### 2.1. Study Design and Rationale 

The study is a secondary longitudinal analysis of the results of a randomized controlled trial (RCT) [18]. ClinicalTrials.gov ID: NCT01449760. The RCT was a single blinded, randomized controlled trial with two intervention groups and one control group: 1) conventional physical therapy; 2) exercise training with the Wii Fit™ balance board (Nintendo, Kyoto, Japan); and 3) no exercise therapy. The hypothesis was that patients using the Wii Fit™ would achieve better results in self-reported physical function of the ankle than patients receiving physical therapy. The primary outcome was self-reported physical function measured with the Foot and Ankle Ability Measure questionnaire. The superiority of the balance board intervention was not supported by the results [18]. 

The motivation for this study was to harness the data of the RCT to better analyze the outcome of the patients. Given that RCT imposed well-defined therapies to participants over six weeks, we found it interesting to assess the long-term recovery and its association with early clinical measures. The idea was to find relevant markers for chronicity.

### 2.2. Participants 

Between April 2010 and July 2014, 90 patients with acute lateral ankle sprains were recruited from the emergency department of the University Hospital of Geneva in Switzerland. The inclusion criteria covered adults aged between 18 and 65 years with grade I or II ankle sprains [12]. Patients were to be excluded if they had a grade III ankle sprain, a fracture, any neurological, musculoskeletal or other disorder that could influence the functionality of the ankle, had undergone active functional therapy during the first four weeks following the sprain, or had already experienced a previous ankle sprain on the same side as the current sprain within the preceding 12 months.

### 2.3. Procedure

The included patients were discharged from the emergency department with semi-rigid Aircast ankle braces and standard painkillers. The physician provided standard instructions regarding the PRICE protocol and recommended only performing pain-free movements for four weeks [9]. Patients wore their ankle braces day and night for three weeks and, if needed, three additional weeks during the daytime only.

Four weeks after injury, the patients attended appointments with an experienced physical therapist who took clinical measurements suspected to serve as predictors of recovery. The physician also recorded the patients’ self-reported pain and self-reported physical functioning (see below for further details concerning the measured predictors). The decision to assess the patients’ conditions at this time was guided by previous results, which showed that clinical assessment administered at four weeks could serve as predictors of recovery [19]. Further information about the treatments can be found in [18]. After the baseline assessment, the patients were randomly assigned to conventional physiotherapy, exercises with the Wii Fit^TM^, or no therapy [18]. Conventional physiotherapy consisted of joint mobilization, muscle strengthening, and proprioceptive exercises. For six weeks, patients participated in nine 30-min sessions and were advised to practice home exercises. Exercises with the Wii Fit™ were instructed by a physical therapist in an initial session. Then, the patients practiced these exercises independently at home, at least two times per week for 30 min per session, over a six-week period. Finally, patients who received no therapy did not receive any exercise therapy. 

Self-reported physical function of the ankle was reassessed seven months after injury.

### 2.4. Outcome Assessment

The main outcome (dependent variable) was whether the study participants were healed from their ankle sprain seven months after the injury. Following the recommendation of Wikstrom [20], patients were considered to have fully recovered if they scored 99% or greater on the foot and ankle ability measure (FAAM) activities of daily life (ADL) score and 97% or greater on the FAAM sport score. Recovery status was used as a two-level categorical variable (recovered or not recovered).

We assessed the different predictors of recovery as follows (further information about the measurement methods can be found in [18]):

- **Edema**: To assess edema (joint swelling), we measured circumference (tape measure) of the ankle at the level of both malleoli and foot circumference, midway between the lateral malleolus and the base of the second metatarsal [21]. These measurements were repeated for both healthy and injured ankles. The selected values were the percent differences between sides;
- **Joint mobility**: Passive ankle ROM was measured with a manual goniometer (GIMA S.p.A, Gessate (MI), Italy). Both plantar and dorsal flexion were tested. Dorsal flexion was measured in both an extended knee position and a 90° flexed knee position [22]. These measurements were repeated for both healthy and injured ankles. The selected values were the percent differences between sides;
- **Muscle strength**: The maximum isometric muscle strength of the ankle’s plantar and dorsal flexor, invertor, and evertor was measured with a handheld dynamometer (microFET2 Hoggan Scientific, Salt Lake City, UT, USA). The foot was placed in a neutral position, and the examiner held the dynamometer stationary while the patient exerted maximal force against it. The dynamometer was positioned as recommended by Spink et al. [23]. Each measurement was taken three times and the maximum result was recorded. These measurements were repeated for both healthy and injured ankles. The selected values were the percent differences between sides;
- **Self-reported pain:** Pain was evaluated by means of a visual analogue scale (VAS) both at rest and while walking [24]. Each patient marked a point on a 10-cm line, ranging from zero for “no pain” to ten for “severe pain”;
- **Self-reported physical function of the ankle:** Ankle function was evaluated with the FAAM questionnaire, which is a valid and reliable self-report questionnaire for patients with foot and ankle disorders [25,26]. This questionnaire consists of 21 items concerning ADL and eight items concerning sports activities. The final score is represented as a percentage from 0 to 100, and a higher score indicates a higher functional level. The minimal clinically important difference is eight points for the ADL subscale and nine points for the sport subscale.

Overall, 13 predictors were used as independent continuous variables. Body mass index (BMI), and sex were also included as covariates. The RCT results showed that the type of rehabilitation had no influence on the patients’ recovery statuses. For the sake of completeness, we still included rehabilitation type in the analysis as a three-level categorical variable.

### 2.5. Statistics

Owing to the high number of potential predictors of ankle sprain recovery, we preselected the most relevant predictors through a random forest (RF) approach [27]. The aim was to reduce the number of predictors to below five to avoid overfitting. An RF is a supervised machine-learning algorithm that aggregates many decision trees to perform classification or regression tasks. RFs offer the ability to determine which predictors are the most important in realizing a classification (variable importance measure, VIM). We used the *Party* (v. 1.2-2) *R* (v. 3.3.3) package and the *cforest* function [28,29,30] to aggregate 3000 conditional inference trees. For VIM computation, we used the conditional implementation (function *varimpAUC)* [31,32,33]. The RF learned to classify patients (recovered patients coded as 1; unrecovered patients coded as 0) based on the 16 predictors (Table 1 and Table 2). Prediction accuracy (kappa) was assessed with out-of-bag (OOB) data, which limited the risk of overfitting. 

The most important predictors, preselected via RF, were then applied as independent variables in logistic regressions, with recovery status as the dependent variable. Both simple and multivariate regressions were evaluated, including interactions. Model selection was carried out using the Bayesian information criterion (BIC), which penalizes model complexity. Discriminative performance was estimated by means of a receiver operating characteristic (ROC) curve and the area under the curve (AUC) with 95% confidence intervals (CI) computed by bootstrapping. The odds ratio of the best model was computed to evaluate effect size. The significance level (type I error rate) was set to 0.05.

## 3. Results

Among the 90 patients that participated in this RCT, 52 completed the seven-month follow up. Reasons for dropping out included: geographical reasons (n = 4), pregnancy (n = 1), surgery unrelated to the ankle sprain (n = 3), new ankle sprain (n = 2), disinterested in continuing (n = 1), holidays (n = 1), sickness due to other disease (n = 2), the time-consuming nature of the follow-up (n = 4), and unknown reasons (n = 20).

Among the 52 participants who attended the seven-month follow-up, 27 patients recovered (52%) and 25 patients did not (48%). The means and standard deviations of the demographic factors are presented in Table 1. The assessed predictors are presented in Table 2. We used medians and interquartile ranges to show variable distributions because we observed some non-normal distributions.

**Figure 1:**
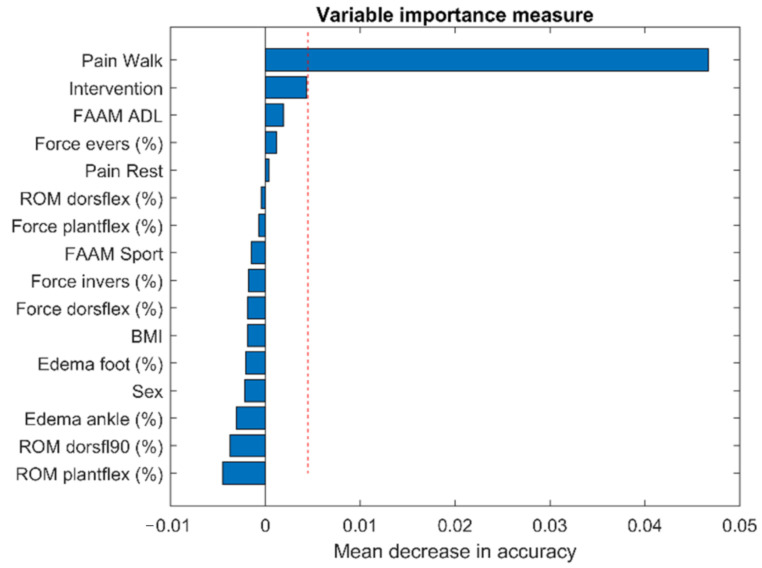
Variable importance measure (VMI) from the random forest model. 3000 decision trees were used. VIM was computed by permutation. The vertical dotted red line highlights the minimal value that can be considered as more important than a random predictor. Pain Walk: Visual Analogue Scale while walking. Intervention: physical therapy, Wii fit™, no treatment. FAAM ADL: FAAM activities of daily living subscale. Force evers: eversion strength. Pain Rest: Visual Analogue Scale during rest. ROM dorsflex: range of motion dorsiflexion. Force plantflex: plantarflexion strength. FAAM Sport: FAAM sport subscale. Force invers: inversion strength. Force dorsflex: dorsiflexion strength. BMI: Body Mass Index. Edema foot: foot edema. Edema ankle: ankle edema. ROM dorsfl90: range of motion dorsiflexion with the knee flexed (90°). ROM plantflex: range of motion plantarflexion.

Due to three missing pieces of data, only 49 participants were included in the RF analysis. The RF analysis correctly classified 62% of the participants. The kappa coefficient was 0.24, corresponding to a poor agreement [32]. The VIM results (Figure 1) identified pain during walking (VASwalking) as the most important variable in predicting recovery. The second most important predictor of recovery was the type of intervention, but the importance of this variable was near the limit of random variation. 

**Figure 2:**
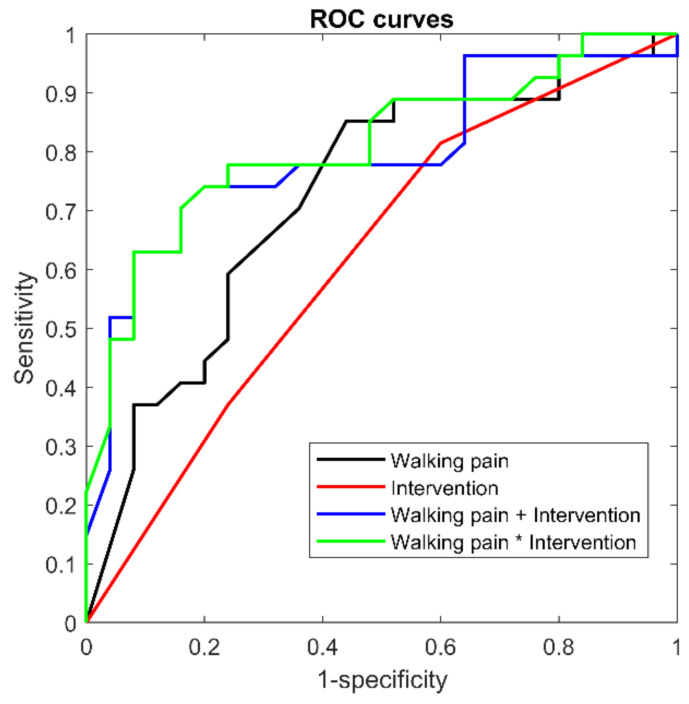
ROC curve with pain while walking four weeks after injury as a predictor for recovery after seven months. The four curves refer to the four models presented in Table 3. ROC: receiver operating characteristic.

Given that two predictors were considered important according to the VIM analysis, four logistic models were tested on the full dataset (n = 52, no missing data). The univariate model, including only pain while walking, exhibited the lowest BIC and an AUC of 0.73 (Table 3 and Figure 2). Multivariate models (with and without interactions) exhibited better predictive power (AUC 0.79 and 0.81), but higher BICs indicate substantial overfitting (Table 3). 

The best model indicated that pain while walking was negatively associated with the probability of recovery (odd ratio: 0.71, 95% CI: 0.53–0.95). In other words, the odds of recovery seven months after the sprain decreased by 29% for each additional point on the pain scale assessed four weeks after the injury.

## 4. Discussion

This study aimed to assess the prognostic value of objective clinical measures (edema, ROM, and muscle strength), self-reported pain, and functional scores assessed four weeks after an ankle sprain to predict recovery at seven months. 

First, we observed that 48% of the study participants did not fully recover from their ankle sprains seven months after their accidents. Comparable rates of poor long-term recovery have been reported in several studies [15,34,35]. In addition, we observed that recovery status was independent of sprain severity (see Table 1; 67% of grade I sprains in the recovered group vs. 60% of grade I sprains in the not-recovered group; not a significant difference χ^2^ = 0.27, *p* = 0.60). The independence of recovery status from the severity of the initial sprain has been attested by a previous systematic review [15]. 

The results of the present study suggested that perceived pain during walking is associated with long-term recovery. This finding is in line with those of previous studies, which have evidenced an association between limitations in weight-bearing activities and disability duration [36], as well as an association between athlete’s ambulation statuses and the predicted number of days to return to sport [37]. Similarly, pain during weight-bearing dorsiflexion assessed four weeks after an ankle sprain has previously been found to be a predictor of recovery at four months [19].

Another study has suggested that recovery can be predicted based on walking ability immediately after the injury [38]. However, other researchers have observed no relationship between pain during weight bearing (evaluated some days after the ankle sprain and then three months after injury) and recovery status at 12 months [34].

At first sight, patient’s pain levels while walking were low. On average, participants who fully recovered scored 1.6 on the 0–10 VAS scale, while participants who did not fully recover scored 3.3 (Table 2). However, there was substantial variability underlying this finding. Nine subjects in the recovered group (33%) reported no pain at all, while only two patients (8%) in the unrecovered group reported zero pain. In contrast, approximately 25% of the unrecovered group and only 11% of the recovered group reported pain levels higher than five. This finding illustrates the substantial heterogeneity of clinical course following acute ankle sprain, as has been highlighted by other researchers [15].

In contrast with self-reported pain, functional capacity assessed via the FAAM questionnaire did not reveal itself as a predictor of recovery. The results of three previous studies contrast with this finding. Firstly, Cross et al. observed that the results of the short form-36 physical functioning scale (SF36PF) correlated with the number of days to return to sport [37]. Secondly, de Bie et al. reported that two- and four-week recovery can be predicted using the function score, combined with the doctor’s opinion and the palpation score [38]. Finally, van der Wees et al. found that the ankle function score can be used to predict ankle sprain outcomes, albeit with low specificity and sensitivity [39]. 

Four weeks after their sprain, the study’s participants still experienced substantial reduction in strengths and ranges of motion, as well as residual edema (Table 2). However, we observed that these objective clinical measures were not retained as recovery predictors (Figure 1); therefore, these measures do not appear to help in estimating long-term (seven months) healing. In contrast, other studies have evidenced that clinical measures (swelling and ROM) measured soon after sprain occurrence are associated with short-term healing [36,38]. This discrepancy underlines the importance of the time at which a patient is assessed. From a clinical perspective, delayed assessment may be preferred. Indeed, during the first weeks after a sprain, the PRICE protocol guides the treatment. Later, assessment of the patient’s status may help in prescribing appropriate rehabilitation. 

The physio-pathological mechanisms of ankle-sprain recovery that lead to either rapid healing or CAI are largely unknown. Recent studies have suggested that successive cascades of sensorimotor reorganization may occur in this process [20]. Patients with CAI have shown high neuromuscular mechanosensitivity in the muscles and nerves surrounding the ankle joint, potentially indicating central sensitization [40]. Other researchers have evidenced that psychosocial factors (adaptiveness in response to pain in particular) may explain the variation in symptoms and limitations that occurs after ankle sprains [41]. In patients recovering from isolated musculoskeletal extremity injuries (fractures or sprains), a higher risk of developing chronic pain was associated with age over 40 years, poor physical health, pain catastrophizing, high urgency level, and severe pain at discharge [42]. Therefore, walking pain persisting after the acute phase of recovery may be a red flag for patients at risk of poor recovery and chronicity: first, it may indicate that underlying sensorimotor reorganisation and central sensitization are underway; second, persistent pain may be bidirectionally associated with detrimental psychological effects (pain catastrophizing, fear-avoidance [43], anxiety, and depression), which may diminish a patient’s motivation and undermine the efficacy of ongoing rehabilitation. 

A strong point of the present study is its external validity. The present study sample included participants representing the general population. All the participants were included in a monocentric RCT, and therefore received uniform care. In addition, all the examined predictors were easily measurable and validated variables, and a clear definition was used for the seven-month recovery status. Finally, we used statistical methods that minimized the risks of overfitting.

Our study had some limitations. First, the sample size was dependent on the size of the parent RCT. Given the large inter-individual variability of the sample (Table 1 and Table 2), and the known heterogeneity in ankle sprain outcomes, a larger sample would be necessary to obtain firmer conclusions. Second, a substantial number of participants were lost to follow-up; this may have biased the results. Finally, the seven-month interval for assessing full recovery was short in comparison to the 12-month interval often used by other long-term follow-up studies.

## 5. Conclusions

In summary, the present study highlighted that (1) nearly half of the patients who underwent lateral ankle sprain had not fully recovered seven months after their sprains, and (2) pain while walking, assessed four weeks after lateral ankle sprain, may help in predicting recovery at seven months. These findings confirm the substantial heterogeneity of the clinical course of ankle sprain. These findings also highlight the importance of the assessment of pain while walking in detecting patients at risk of poor long-term prognosis. Assessing patients after the acute phase of ankle sprain recovery may help in selecting ideal rehabilitation treatments. Particular attention should be paid to patients with persistent pain in order to reduce the risk of chronicity.

## Figures and Tables

**Table 1:** Descriptive statistics: patients’ characteristics.

Variables	Recovered (n = 27)		Not Recovered (n = 25)	
	n	Mean (SD) or %	n	Mean (SD) or %
Age (year)	27	34 (9)	25	36 (12)
BMI (kg/m^2^)	27	26.2 (4.3)	25	26.0 (4.3)
Sex	27		25	
Male	16	59%	13	52%
Female	11	41%	12	48%
Severity	27		25	
Grade I	18	67%	15	30%
Grade II	9	33%	10	40%
Previous sprain	27		24	
Yes	16	59%	16	67%
No	11	41%	8	33%
Sport practice	27		25	
Yes	23	85%	19	76%
No	4	15%	6	24%
High-load sport	27		24	
Yes	14	52%	11	46%
No	9	48%	13	54%

SD: standard deviation.

**Table 2:** Descriptive statistics: recovery predictors.

Measurements	Recovered (n = 27)		Not Recovered (n = 25)	
	n	Median (1st and 3rd Quartiles) or %	n	Median (1st and 3rd Quartiles) or %
Edema (%)				
Ankle	27	4.1 (−3.0 – 10.6)	24	2.1 (−3.9 – 8.8)
Foot	27	2.0 (−4.1 – 8.5)	24	2.1 (−6.0 – 6.3)
Range of motion (%)				
Dorsiflexion	25	−16.7 (−33.3 – −6.9)	25	−25.0 (−60.0 – 0.0)
Dorsiflexion 90°	27	−16.7 (−27.6 – −4.9)	25	−20.0 (−40.0 – −9.7)
Plantar flexion	27	−16.7 (−22.0 – −5.6)	25	−9.1 (−18.4 – 0.0)
Strength (%)				
Dorsiflexion	27	−20.0 (−27.1 – −2.3)	25	−13.3 (−33.7 – −7.2)
Plantar flexion	27	−14.0 (−20.3 – −1.6)	25	−15.3 (−31.9 – −1.5)
Inversion	27	−9.9 (−43.6 – −2.5)	25	−12.8 (−43.2 – −5.4)
Eversion	27	−22.1 (−37.1 – −6.9)	25	−15.4 (−23.0 – −2.7)
Pain (VAS 0–10)				
Rest	27	0.2 (0.0 – 1.0)	25	0.5 (0.0 – 2.0)
Walking	27	1.1 (0.1 – 2.0)	25	2.4 (1.5 – 4.9)
Ankle function (0–100)				
FAAM ADL	27	86.9 (71.9 – 94.6)	25	73.8 (61.9 – 86.3)
FAAM Sport	27	46.9 (28.1 – 72.5)	25	40.6 (12.5 – 56.3)
Intervention				
Physical therapy	12	44%	9	36%
Wii fit™	5	19%	10	40%
No treatment	10	37%	6	24%

Participants’ assessment took place four weeks after ankle injury. For oedema, range of motion and strength, the values were calculated as percent difference between injured and uninjured sides. VAS: visual analog scale. FAAM: foot and ankle ability measure. ADL: activities of daily life.

**Table 3:** Logistic regressions.

n = 52	Model	BIC	AUC	AUC CI	
Simple regressions	walking pain	72.9	0.73	0.57	0.85
	intervention	80.8	0.62	0.45	0.75
Multivariable regressions	without interaction	77.1	0.79	0.65	0.90
(pain and intervention)	with interaction	79.0	0.81	0.66	0.91

BIC: Bayesian information criterion. AUC: area under the ROC curve. CI: 95% confidence interval.

## Data Availability

The data presented in this study are available from the corresponding author on reasonable request. The data are not publicly available due to the non-consent of the study participants for public disclosure of their medical information, even in an anonymized form.
